# Research Advances in the Immunomodulatory Functions of CD100/SEMA4D and Their Roles in Viral Infectious Diseases

**DOI:** 10.3390/ijms26094341

**Published:** 2025-05-02

**Authors:** Mengxiao Zhao, Liwei Chen, Yuhang Chen, Xuecheng Yang, Xuemei Feng, Dongliang Yang, Xin Zheng, Jia Liu

**Affiliations:** 1 Department of Infectious Diseases, Union Hospital, Tongji Medical College, Huazhong University of Science and Technology, Wuhan 430022, China; zhaomengxiao@hust.edu.cn (M.Z.);; 2 Institute of Infectious Diseases and Immunity, Union Hospital, Tongji Medical College, Huazhong University of Science and Technology, Wuhan 430022, China; 3 Joint International Laboratory of Infection and Immunity, Huazhong University of Science and Technology, Wuhan 430022, China

**Keywords:** CD100/SEMA4D, viral infection, immunomodulatory, immune responses

## Abstract

CD100/SEMA4D, a member of the Semaphorin family, is a transmembrane glycoprotein that regulates neurogenesis, immune modulation, and angiogenesis, with its immunoregulatory roles having attracted considerable attention. It is dynamically expressed on the surface of diverse immune cells—including T cells, B cells, dendritic cells (DCs), and natural killer (NK) cells—with expression levels modulated by cellular activation states. CD100 exists in two functional forms: membrane-bound CD100 (mCD100) and soluble CD100 (sCD100) generated via proteolytic cleavage. Recent studies have highlighted its critical involvement in viral infectious diseases. This review systematically summarizes the molecular characteristics, expression patterns, and regulatory functions of CD100 on different immune cells, and discusses its role in viral infectious diseases and its clinical application potential.

## 1. Introduction

CD100 (also known as Sema4D), a member of the Semaphorin family, regulates nervous system development and synapse formation. Emerging evidence over recent decades has revealed that several Semaphorin family members significantly participate in immune cell activation and immunomodulation, among which CD100 represents the first identified Semaphorin protein with immunoregulatory functions [1,2,3]. Accumulating studies have demonstrated the pivotal immunomodulatory roles of CD100 in various pathological conditions, including anti-infection immunity, autoimmune disorders, and tumor immune microenvironment regulation [4,5,6]. Although the immune-regulatory properties of CD100 have been extensively investigated, its precise molecular mechanisms and context-dependent functions in different immunological settings remain unclear. Furthermore, the therapeutic potential of CD100 as an immunomodulatory target warrants in-depth exploration. This review systematically synthesizes current knowledge regarding the structural characteristics, expression patterns, and functional diversity of CD100 across various immune cell populations, with particular emphasis on its involvement in viral infection-related pathologies (Table 1). Additionally, we evaluate the translational potential of CD100-targeted immunotherapy and propose future research directions to advance this evolving field. By integrating existing findings and identifying knowledge gaps, this comprehensive analysis aims to provide valuable insights for subsequent investigations into CD100-mediated immunoregulation.

## 2. Structural Features and Expression Patterns of CD100

**Figure 1:**
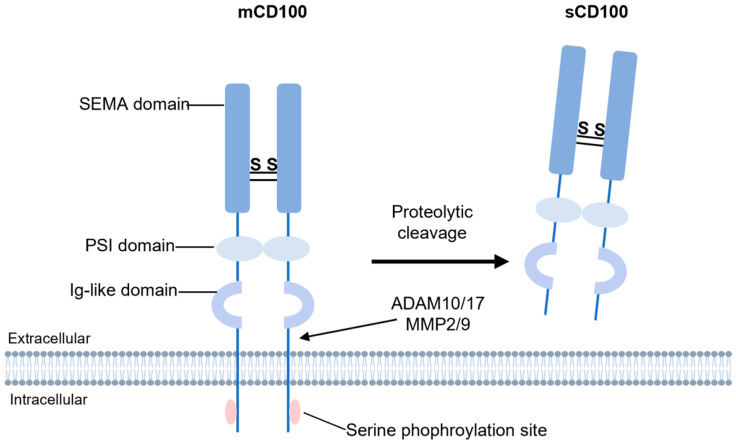
The structure of CD100. The structure of mCD100 consists of cytoplasmic serine phosphorylation sites, a transmembrane domain, an extracellular region containing an Ig-like domain, a PSI domain, and a SEMA domain. The membrane-bound form can be proteolytically processed by ADAM10/17 and MMPs to generate soluble CD100. Ig, immunoglobulin; PSI, Plexin-semaphorin-integrin; MMPs, matrix metalloproteinases.

CD100 is a transmembrane glycoprotein with a molecular weight of 150 kDa, consisting of 862 amino acids [16]. Its structure includes three primary domains: a cytoplasmic region containing serine phosphorylation sites, a transmembrane domain, and a large extracellular region composed of multiple conserved structural motifs (Figure 1). The extracellular domain features a SEMA domain (approximately 500 amino acid residues in length), a hallmark of the Semaphorin family responsible for receptor binding, along with an immunoglobulin (Ig)-like domain and a conserved Plexin-semaphorin-integrin (PSI) domain [17,18]. The transmembrane domain anchors the extracellular region in the cell membrane. The relatively short intracellular region suggests that it primarily transmits signals through interactions with other signaling molecules. Proteolytic cleavage of the extracellular domain by enzymes such as ADAM10 and matrix metalloproteinases (MMP2/9) generates a soluble 120 kDa form (sCD100) [19,20], which acts as a ligand to mediate immunomodulatory functions. Most studies have focused on sCD100 [7,21,22,23], whereas the biological significance of membrane-bound CD100 (mCD100) remains less explored, despite its critical roles.

CD100 is widely expressed in diverse tissues and cell types. Within the immune system, it is highly expressed on resting T cells but shows lower baseline expression in antigen-presenting cells (APCs), including B cells and dendritic cells (DCs) [24,25,26]. Upon activation, these cells rapidly upregulate CD100 expression [27]. Additionally, CD100 is detectable in natural killer (NK) cells and monocytes, where it regulates their migration and effector functions [11,26,28]. Beyond the immune system, CD100 contributes to neuronal axon guidance and synaptogenesis in the nervous system. In tumor tissues, its expression correlates with invasiveness and clinical prognosis [5]. The structural versatility of CD100 enables interactions with multiple receptors, allowing it to orchestrate immunoregulation, neurodevelopment, and tumor progression.

CD100 binds two major receptor classes: high-affinity Plexin family members (Plexin-B1 and Plexin-B2) and low-affinity CD72 [27,29]. CD72 is predominantly expressed in lymphoid organs (e.g., lymph nodes and spleen) and immune cells, including T cells, B cells, DCs, and NK cells [30]. The classical paradigm posits tissue-specific receptor utilization: CD100 primarily engages Plexin family receptors (e.g., Plexin-B1/B2) in non-immune tissues to mediate processes such as axon guidance in the nervous system and endothelial cell migration in the cardiovascular system, whereas CD72 drives immune-specific functions including T/B cell activation and DC maturation in lymphoid tissues [27,31,32,33,34]. However, emerging evidence challenges this view. Plexin receptors have been identified in immune cells [28,35], and CD72 expression has been detected in non-immune contexts, particularly on neuronal membranes [36]. These findings enable a more comprehensive interpretation of previous research data on CD100’s regulatory functions in both neural and immune regulation.

## 3. Regulatory Roles of CD100 in Immune Cells

### 3.1. T Cells

**Figure 2:**
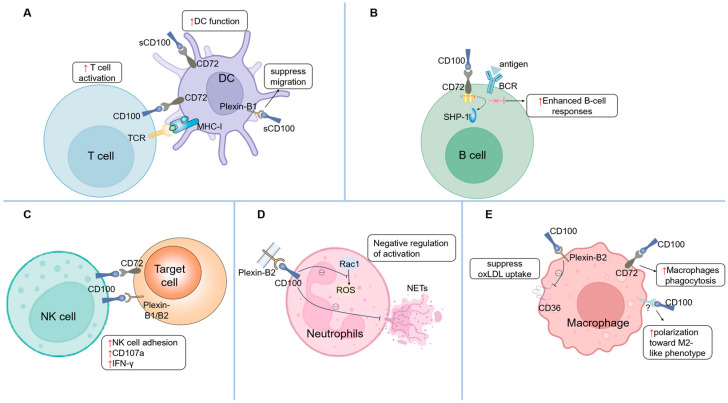
Different functions of CD100 in multiple immune cells. (**A**) CD100 enhances T cell activation through both direct mechanisms and indirect pathways involving DC-mediated regulation. CD100 promotes the activation and maturation of DCs via binding to CD72 and inhibits its migration by interacting with Plexin-B1. (**B**) CD100–CD72 interaction blocks SHP-1 recruitment to ITIMs, promoting B cell proliferation and antibody production. (**C**) mCD100 binding to CD72/Plexin-B1/B2 on target cells enhances adhesion, degranulation (CD107a), and IFN-γ secretion of NK cells. (**D**) mCD100–Plexin-B2 interaction inhibits Rac1-dependent ROS generation and the formation of NETs. (**E**) CD100–CD72 enhances the phagocytic ability of macrophages against parasites, while CD100–Plexin-B2 downregulates the expression of CD36 and inhibits the uptake of oxLDL. CD100 also promotes M2-like polarization (receptor unknown). SHP-1, SH2-containing protein tyrosine phosphatase-1; ITIMs, immunoreceptor tyrosine-based inhibitory motifs; ROS, reactive oxygen species; NET, neutrophil extracellular trap.

CD100 is constitutively expressed on T cells and serves as a critical molecular bridge facilitating interactions between T cells and other immune cells. Early studies established its essential role in T cell priming and differentiation. In CD100-deficient mice, antigen-specific CD4^+^ T cells in draining lymph nodes show markedly reduced proliferation and cytokine production [37,38]. These mice also exhibit resistance to experimental autoimmune encephalomyelitis (EAE) attributed to defective generation of myelin oligodendrocyte glycoprotein (MOG)-specific T cells [38]. Conversely, CD100-transgenic mice display enhanced T cell responses, further confirming its immunomodulatory potency [39]. Notably, CD100-deficient T cells retain normal responsiveness to CD3 monoclonal antibody or concanavalin A stimulation [37], while recombinant sCD100 fails to directly activate T cells. These paradoxical observations initially suggested that mCD100 on T cells primarily acts as a ligand to promote dendritic cell (DC) maturation, thereby indirectly driving T cell activation. Supporting this, DCs from CD100-deficient mice demonstrate functional impairments and reduced co-stimulatory molecule expression. Importantly, exogenous sCD100 restores DC functionality and rescues T cell responses in these mice [38]. Emerging evidence, however, reveals direct regulatory roles of mCD100 in T cells through CD100–CD72 and CD100–Plexin-B2 pathways. Engagement of mCD100 with CD72 or Plexin-B2 induces phosphorylation of Lck/ZAP70, triggering early T cell activation signals [40]. In unstimulated T cells, mCD100 initiates TCR-like signaling, whereas it functions as a co-stimulatory molecule in activated T cells. During HCV infection, IFN-α enhances CD100 expression on naïve CD8^+^ T cells via the CD100–CD72 axis, amplifying antiviral immunity [10]. Functional studies further demonstrate that anti-CD100 monoclonal antibodies or soluble CD72 blockers suppress T cell proliferation, while CD72-Fc reverses this inhibition [41]. Activated T cells upregulate CD72 expression, suggesting bidirectional signaling capacity of endogenous CD100—acting both as a receptor and a ligand through CD72 interactions. Collectively, these findings demonstrate that CD100 orchestrates T cell activation through both direct mechanisms (mediated by CD100–CD72/Plexin-B interactions) and indirect pathways involving DC-mediated regulation (Figure 2A).

Studies have shown that the CD100–Plexin-B2 pathway in γ δ T cells is essential for skin wound healing in epithelial tissues [42]. CD100-deficient γ δ T cells display reduced responsiveness to keratinocyte injury, resulting in the delayed repair of dermal wounds. Within the wound microenvironment, Plexin-B2, expressed by keratinocytes, binds to CD100 on activated γ δ T cells. This interaction triggers cellular morphological changes through downstream extracellular signal-regulated kinases (ERK) phosphorylation and cofilin-mediated actin remodeling. Together, these signaling cascades drive cellular morphological changes that are critical for efficient wound closure.

### 3.2. B Cells

Current evidence highlights the critical regulatory role of the CD100/CD72 signaling pathway in B cell activation and functional maturation. Previous studies demonstrate that the interaction between CD100 and CD72 on B cell surfaces significantly enhances cellular responsiveness. Mechanistically, CD72 contains intracellular immunoreceptor tyrosine-based inhibitory motifs (ITIMs) that recruit SH2-containing protein tyrosine phosphatase-1 (SHP-1), thereby suppressing downstream signaling pathways (e.g., Akt/NF-κB and p-65/ERK) during B cell receptor (BCR) activation [43,44,45]. CD100 engagement triggers ITIM dephosphorylation, leading to SHP-1 dissociation and the subsequent activation of these pathways, ultimately promoting B cell activation and antibody production [20,27]. Notably, CD100-deficient mice exhibit impaired antibody responses to T cell-dependent antigens [37]. Splenic B cells from these mice display reduced proliferation upon CD40 monoclonal antibody stimulation, a defect reversible by CD72 monoclonal antibody treatment [46]. In vitro studies further confirm that CD100 enhances B cell aggregation, proliferation, survival, and CD40-CD40L signaling through CD23 downregulation [1,27]. The CD100/CD72 axis also regulates B cell subpopulation development. CD5^+^ B-1 cells are markedly reduced in CD100-knockout mice [37], whereas CD100 overexpression expands CD5^+^ B-1 populations without affecting conventional B or T cell numbers, while augmenting B cell proliferation and immunoglobulin production [39]. Additionally, Plexin-B1 on murine epithelial cells interacts with mCD100 on CD5^+^ B cells to enhance proliferation and suppress apoptosis [25]. Collectively, these findings establish that CD100 modulates B cell development, activation, and function through dual interactions with CD72 and Plexin-B1 (Figure 2B).

### 3.3. Dendritic Cells (DCs)

Previous research has established that CD100 primarily regulates dendritic cells (DCs) maturation and functionality through its interaction with CD72. Kumanogoh et al. [38] demonstrated that DCs from CD100-deficient mice exhibit impaired interleukin-2 (IL-2) secretion [47], while recombinant sCD100 administration enhances surface expression of co-stimulatory molecules (CD80/CD86) and MHC class II, along with augmenting CD40-stimulated immunogenicity. As discussed in the “T cells” section, CD100 expressed on T cells actively promotes DC activation and maturation through cellular interactions. Furthermore, emerging evidence reveals that soluble sCD100 suppresses both spontaneous and MCP-3-induced migration of myeloid-derived immature DCs. This inhibitory effect has been mechanistically linked to sCD100 binding with Plexin-B1 receptors on DC surfaces [28], highlighting a novel regulatory pathway distinct from its canonical CD72-mediated signaling (Figure 2A).

### 3.4. Natural Killer (NK) Cells

Emerging evidence delineates the functional role of CD100 expression on activated natural killer (NK) cells in target cell cytotoxicity, mediated through dual receptor interactions. While CD100 does not directly participate in NK cell-killing mechanisms, it operates as an adhesion modulator that enhances NK cell-target cell conjugation, thereby potentiating cytotoxic efficacy and interferon-gamma (IFN-γ) secretion, as demonstrated in prior studies [26]. Mechanistically, CD100 engages CD72 on target cells to facilitate this adhesion-dependent functional augmentation. Recent investigations by He et al. [11] reveal an additional dimension of CD100 signaling: engagement of Plexin-B1/B2 receptors on target cells by mCD100 on NK cells significantly amplifies degranulation and IFN-γ production. Notably, this Plexin-mediated activation is competitively inhibited by sCD100, which disrupts receptor–ligand binding through molecular interference (Figure 2C).

### 3.5. Neutrophils

In contrast to its roles in other immune cells, CD100 exerts negative regulatory effects on neutrophil activation. Mechanistically, CD100 on neutrophil surfaces interacts with Plexin-B2 to suppress reactive oxygen species (ROS) generation and attenuate neutrophil extracellular trap (NET) formation. Recombinant Plexin-B2 inhibits the activation of Rac1—a small GTPase critical for ROS production—through CD100’s intracellular domain (Figure 2D). Notably, formyl-methionyl-leucyl-phenylalanine (FMLP)-induced shedding of mCD100 partially reverses this suppressive effect [48].

Early investigations revealed that sCD100 inhibits both spontaneous and MCP-3-induced migration of human monocytes and U937 monocytic cells. These inhibitory effects were abolished by anti-CD100 monoclonal antibodies, though the receptor mediating this phenomenon remained unidentified in initial studies [49]. Subsequent work demonstrated that sCD100-mediated suppression of monocyte spontaneous migration is abolished upon the Plexin-C1 blockade [28]. While these findings implicate Plexin-C1 in transducing CD100’s inhibitory signals, direct biochemical evidence confirming physical interaction between CD100 and Plexin-C1 remains elusive [29].

### 3.6. Macrophages

CD100 exerts multiple immunoregulatory effects on macrophages through interactions with its receptors, CD72 and Plexin B1/B2 [50]. sCD100 demonstrates dual regulation of phagocytic activity. While suppressing oxidized low-density lipoprotein (oxLDL) uptake by downregulating scavenger receptor CD36 via Plexin-B2 signaling [51], it augments phagocytosis of parasites by macrophages in a CD72 receptor-dependent manner [52]. CD100 also promotes macrophage polarization toward an anti-inflammatory M2-like phenotype, characterized by increased IL-10 secretion and reduced production of pro-inflammatory cytokines (IL-6, IL-8, TNF-α), accompanied by elevated expression of the M2 marker CD163 [28] (Figure 2E). Furthermore, CD100 modulates inflammatory signaling by reducing NF-κB activation and reactive oxygen species (ROS) production, effectively eliminating pathogens while preventing host tissue damage from excessive immune responses, thereby maintaining a balance between immune defense and tissue homeostasis [28,52].

## 4. CD100 in Viral Infectious Diseases

The immune system plays a pivotal role in counteracting viral infections through the orchestrated interplay of innate and adaptive immunity, achieving precise pathogen clearance while maintaining immune homeostasis. However, patients with chronic viral infections frequently exhibit compromised T cell responses, often progressing to an exhausted state characterized by diminished viral clearance capacity [53,54]. Recent advances have positioned CD100 as a critical immunoregulator in viral pathogenesis. Emerging evidence has illuminated its multifaceted immunomodulatory functions across diverse viral infections, which will be systematically analyzed in this section (summarized in Table 1).

### 4.1. Hepatitis B Virus (HBV)

The regulatory role of the CD100/CD72 in HBV infection and its impact on antiviral T cell responses were first systematically investigated by Yang et al. [7] Investigations revealed that sCD100 levels were markedly elevated during acute self-limited HBV clearance, whereas chronic hepatitis B (CHB) patients exhibited significantly lower peripheral sCD100 concentrations compared to healthy controls. In vitro and in vivo administration of sCD100 robustly enhanced the expression of co-stimulatory molecules (CD80/CD86) and interleukin-12 (IL-12) secretion in antigen-presenting cells (APCs), particularly dendritic cells (DCs). In HBV-replicating murine models, recombinant sCD100 treatment substantially augmented intrahepatic HBV-specific CD8^+^ T cell responses, thereby accelerating viral clearance. Conversely, the blockade of CD100–CD72 interaction attenuated intrahepatic HBV-specific CD8^+^ T cell activity and delayed viral elimination. These findings establish that the sCD100/CD72 axis critically modulates HBV infection outcomes by fine-tuning virus-specific CD8^+^ T cell responses.

A study in HBV-associated acute-on-chronic liver failure (HBV-ACLF) patients [8] demonstrated that plasma sCD100 levels were significantly lower in HBV-ACLF cohorts compared to asymptomatic carriers, CHB patients, and healthy controls, correlating inversely with disease severity. HBV-ACLF patients exhibited elevated mCD100 expression on CD14^+^ monocytes, accompanied by impaired cytotoxic capacity, reduced IFN-γ/TNF-α/Granzyme B secretion, and compromised monocyte-mediated CD4^+^/CD8^+^ T cell activation. sCD100 treatment restored monocyte functionality by enhancing Granzyme B production, cytotoxic capacity against target cells, and antigen-presenting capacity, while simultaneously revitalizing cytokine secretion in CD4^+^/CD8^+^ T cells. This study delineates a pathological imbalance between sCD100 and mCD100 in HBV-ACLF, which exacerbates immunosuppression through monocyte functional impairment.

### 4.2. Hepatitis C Virus (HCV) 

In chronic hepatitis C virus (HCV) infection, CD100 serves as a pivotal immunoregulatory molecule across multiple immune cell populations. Investigations reveal that B cells in chronic HCV patients exhibit an activated phenotype, characterized by elevated frequencies of CD5^+^ B cells and upregulated expression of CD100, CD69, and CD86 [9]. Notably, IFN-α therapy restores B cell homeostasis in patients achieving sustained virologic responses (SVR), normalizing both CD5^+^ B cell frequencies and activation marker expression. Further research revealed that IFN-α treatment enhances CD100 expression on B cells and subsets in early virologic responders, with CD100 levels inversely correlated with HCV RNA loads, suggesting its potential role in viral control. CD100 interacts with CD72 on B cells, inhibiting the negative regulatory function to promote B cell activation and immune responses. Parallel interactions between CD100 and CD72 on CD8^+^ T cells may similarly potentiate T cell activation.

Subsequent work by the same group demonstrates that HCV infection downregulates CD100 expression on CD8^+^ T cells, whereas IFN-α therapy significantly upregulates CD100 on naïve CD8^+^ T cells. CD100-overexpressing naïve CD8^+^ T cells robustly stimulate IFN-γ and TNF-α secretion in peripheral blood mononuclear cells (PBMCs) and enhance cytotoxicity against HCV-infected cells. These effects require cell–cell contact and correlate with CD72-mediated signaling activation [10].

Recent findings characterize CD100 dynamics in natural killer (NK) cells: HCV infection moderately reduces CD100 expression, while IFN-α therapy markedly upregulates it. In vitro experiments confirm that IFN-α enhances NK cell degranulation (CD107a expression) and IFN-γ secretion by elevating both CD100 on NK cells and Plexin-B1/B2 receptors on target cells, thereby augmenting cytotoxic activity against HCV-infected hepatocytes. Crucially, the blockade of CD100–Plexin-B1/B2 interactions using sCD100 or receptor-specific antibodies abrogates these cytotoxic effects, confirming pathway specificity [11].

A series of studies have proven that CD100 modulates the function of multiple immune cells through interactions with different receptors in HCV infection, thereby enhancing the immune response to HCV. CD100 may also serve as a potential marker for predicting treatment outcomes.

### 4.3. Human Immunodeficiency Virus (HIV)

CD100 functions as a critical immunoregulatory molecule in HIV pathogenesis, engaging in complex immune networks through its receptors, CD72 and Plexin B1. Accumulating evidence indicates that dysregulated CD100 expression patterns correlate strongly with T cell exhaustion and B cell dysfunction in HIV infection. Pioneering work by Eriksson et al. [12] first demonstrated significant downregulation of mCD100 on peripheral CD8^+^ T cells from HIV^+^ individuals. And CD100-deficient cells exhibit impaired effector functions including reduced IFN-γ and TNF-α production. Further analysis revealed co-expression of CD100^−^ CD8^+^ T cells with canonical exhaustion (PD-1) and senescence (CD57) markers, and CD100 expression levels positively correlated with HIV-specific T cell responses. Notably, CD100 depletion is associated with persistent antigen-driven membrane protein shedding, a phenotype persisting despite antiretroviral therapy (ART), indicative of long-term T cell functional impairment.

Extending these findings, Vadasz et al. [13] documented significantly reduced sCD100 levels in HIV^+^ patients compared to healthy controls, with incomplete restoration even after two years of ART. Since sCD100 can bind to CD72 on B cells to relieve negative regulation and promote activation and antibody production, its long-term low levels may worsen B cell dysfunction and insufficient neutralizing antibody production in HIV infection. This CD100–CD72 axis disruption undermines B cell helper function for T cells.

Recent breakthroughs by Correa-Rocha et al. [55] elucidate synergistic effects between CD100/CD72 and PD-1/PD-L1 pathways. HIV^+^ individuals exhibited coordinated upregulation of both axes on T and B cells. CD100 expression intensity on CD4^+^ T cells positively correlated with PD-1/PD-L1 levels, suggesting CD100-mediated T cell activation might indirectly drive exhaustion markers. Strikingly, elevated CD100 on B cells correlated with PD-L1 expression on CD8^+^ T cells, implying bidirectional B-T cell interactions through CD100–CD72/PD-L1-PD-1 pathways that amplify immunosuppressive microenvironments. While ART effectively reduced PD-1/PD-L1 expression, persistently elevated CD72 on T cells post-treatment suggests its potential as an immunological memory marker sustaining dysregulation. Moreover, CD72 expression on CD8^+^ T cells positively correlated with IFN-γ production capacity, revealing context-dependent costimulatory functions that redefine the complexity of CD100–CD72 interactions.

### 4.4. Hantaan Virus (HTNV)

Hantavirus infection can induce hemorrhagic fever with renal syndrome (HFRS), a condition where elevated plasma sCD100 levels during the acute phase correlate with disease severity, likely originating from mCD100 shedding on peripheral blood mononuclear cells (PBMCs) [15]. The authors suggest that sCD100, by binding to CD72 on B cells, may drive their proliferation and antibody production, resulting in immune complex deposition in the kidneys. Moreover, sCD100 may interact with Plexin-B1 on vascular endothelial cells, working with VEGF to boost vascular permeability and worsen vascular damage. Another investigation [14] identified a novel CD8^+^ T cell subset (CD8^low^ CD100^−^ T cells) exhibiting distinct kinetic patterns in HFRS progression. This population was significantly elevated in early-stage patients compared to healthy controls, declined during late-stage disease, and disappeared in convalescence. Characterized by heightened activation and potent cytotoxicity, these cells secreted excessive IFN-γ and TNF-α. In mild cases, the frequency of CD8^low^ CD100^−^ cells among virus-specific CD8^+^ T cells is higher than in severe cases, and their proportion is inversely related to the HTNV viral load. The authors proposed that CD100 deficiency in this subset enhances effector function—a finding that challenges the conventional paradigm of CD100 solely as a co-stimulatory molecule. However, no mechanistic studies were conducted to elucidate the underlying pathways.

## 5. Clinical Prospects of CD100

CD100 emerges as a promising therapeutic target across diverse pathologies, owing to its immunomodulatory roles in autoimmune disorders, viral infections, and tumor microenvironments. Its dynamic expression patterns during disease progression position CD100 as a biomarker for disease prognosis and treatment efficacy assessment.

Current clinical development focuses on Pepinemab (VX15/2503), a humanized IgG4 monoclonal antibody targeting CD100 developed by Fisher et al. [56] Phase I trials in multiple sclerosis (MS) demonstrated favorable tolerability across all dosage levels, with no dose-limiting toxicities reported [57]. Recent Phase II trials data in early-stage Huntington’s disease (HD) revealed not only sustained safety but also therapeutic efficacy, evidenced by improvements in cognitive function, cerebral metabolic activity, and attenuation of brain atrophy and astrocytic reactivity [58,59]. In oncology, a Phase I/II trial evaluating Pepinemab (20 mg/kg administered intravenously every 14 days) in pediatric/adolescent patients with relapsed/refractory solid tumors confirmed target saturation and acceptable safety profiles, though antitumor efficacy requires further optimization.

Beyond therapeutic applications, CD100 exhibits biomarker potential through its dual molecular forms. Elevated plasma sCD100 levels correlate with disease severity in infectious/inflammatory conditions [15,60,61,62], likely reflecting immune activation via proteolytic cleavage of mCD100 on stimulated leukocytes. Overexpression of mCD100 in tumor microenvironment associates with poor prognosis in multiple malignancies [63,64], suggesting utility as a predictive biomarker. However, rigorous validation through large-scale clinical correlative analyses remains imperative to confirm these observations.

## 6. Conclusions

Despite significant advances in understanding CD100 and its regulatory roles across diseases, critical scientific questions remain unresolved. The complexity of CD100’s interactions with multiple receptors and its downstream signaling networks requires systematic elucidation. Notably, how CD100 exerts context-dependent immunomodulatory functions through distinct receptors in varied immune microenvironment remains poorly defined. Furthermore, while current clinical trials of CD100-targeted monoclonal antibodies (e.g., Pepinemab) primarily focus on neurological disorders and cancer, CD100’s potent immunoregulatory properties in viral infections—particularly its capacity to activate immune cells—highlight its untapped potential as a therapeutic target for chronic viral persistence.

## Figures and Tables

**Table 1:** Summary of CD100-mediated immunomodulation in viral infectious diseases.

Infection	Cells Affected	Expression Pattern	Pathway	Effect	References
HBV (Chronic)	CD8^+^ T cell B cell	↑mCD100 ↓sCD100	sCD100/CD72	↓IFN-γ, TNF-α ↓No. of CD100^+^ B cell	Yang et al. [7]
HBV (Acute)	CD8^+^ T cell	↓mCD100 ↑sCD100	sCD100/CD72	↑IFN-γ, IL-2, TNF-α	Yang et al. [7]
HBV (in vitro)	LSEC DC		sCD100/CD72	↓T cell suppression mediated by LSEC ↑CD80, CD86, IL-2	Yang et al. [7]
HBV-ACLF	CD14^+^ monocyte	↑mCD100 ↓sCD100	sCD100/CD72	↓IFN-γ, TNF-α ↓Granzyme B	Zhang et al. [8]
HCV (Chronic)	B cell	↑mCD100			He et al. [9]
	CD8^+^ T cell	↓mCD100	mCD100/CD72	↓IFN-γ, TNF-α ↓Granzyme B, perforin	Li et al. [10]
	NK cell	↓mCD100	mCD100/PlexinB1, B2	↓CD107a ↓IFN-γ	He et al. [11]
HIV	CD8^+^ T cell CD4^+^ T cell	↓mCD100 ↓sCD100	CD100/CD72	↓IFN-γ, TNF-α ↓perforin	Eriksson et al. [12] Vadasz et al. [13]
HTNV	CD8^lo^ CD100^−^ T cell	↑sCD100		↑activation (CD38, HLA-DR) ↑IFN-γ, TNF-α ↑Granzyme B, perforin	Liu et al. [14] Liu et al. [15]

## Data Availability

No new data were created or analyzed in this study. Data sharing is not applicable to this article.
